# Partial correlation analysis for functional connectivity studies in cortical networks

**DOI:** 10.1186/1471-2202-15-S1-P99

**Published:** 2014-07-21

**Authors:** Daniele Poli, Vito Paolo Pastore, Sergio Martinoia, Paolo Massobrio

**Affiliations:** 1 Department of Informatics, Bioengineering, Robotics and Systems Engineering (DIBRIS), University of Genova, Genova, 16145, Italy

## 

The use of *in vitro* neuronal systems and computational models has strongly contributed to the better understanding of relevant neurophysiological principles. Taking advantages form these approaches, here we propose a revised method of partial correlation analysis to investigate functional connectivity in neuronal networks.

The main goal of this work is to estimate the functional connectivity, by means of correlation and information theory-based methods, from the spontaneous activity of dissociated cortical neurons developing *in vitro* and coupled to micro-electrode arrays (MEAs). In particular we focused here on the Partial Correlation (PC) method [1] compared to Transfer Entropy [2] and Cross Correlation (CC) [3] algorithms.

**Figure 1:**
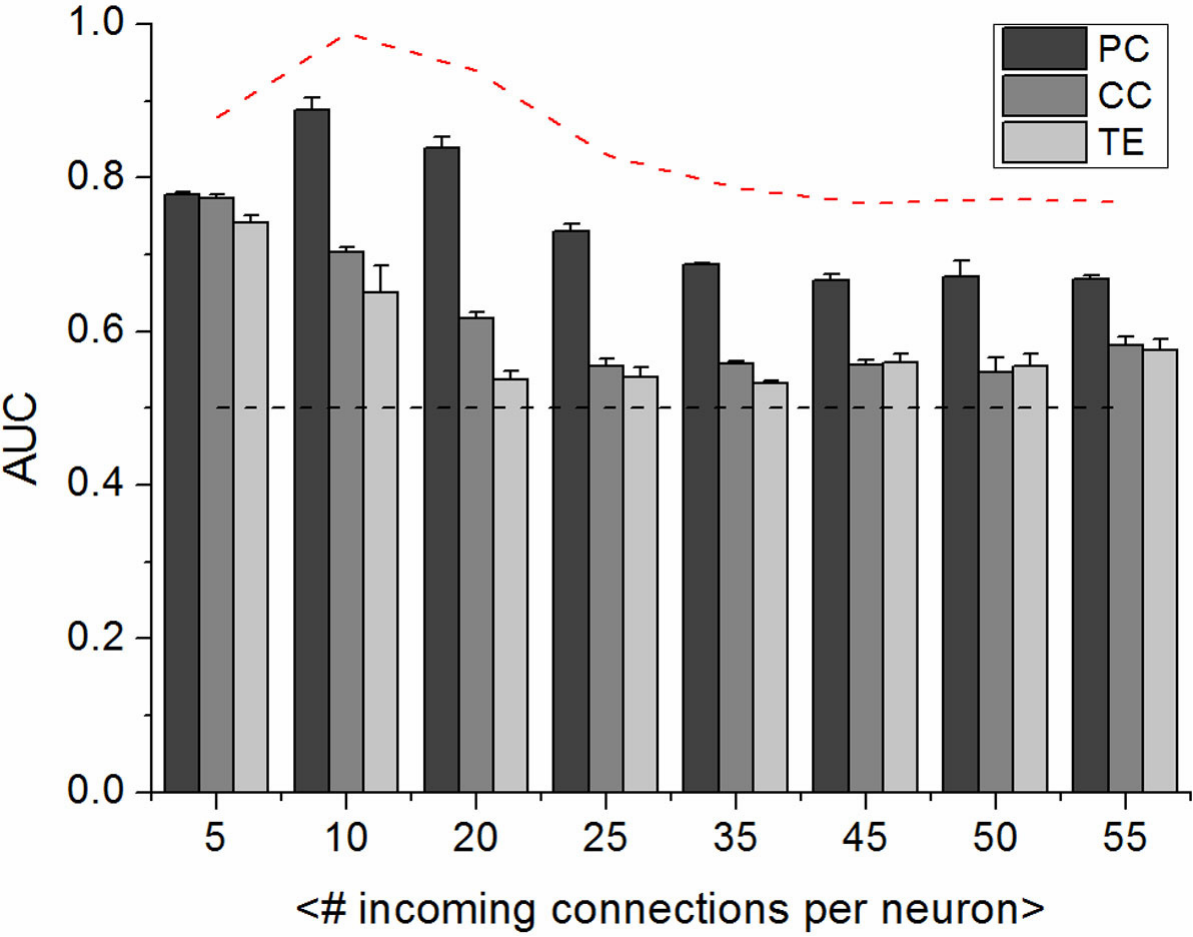
AUC values: Comparison among the three algorithms (PC, CC and TE).

We first evaluated the methods’ performances applying the algorithms to a neural network model made up of 60 spatially distributed and synaptically connected Izhikevich neurons [4]. Using receiver operating characteristic (ROC) curves, calculating the values of the areas under these curves (AUC) and varying the average degree of each cell, we observed that Partial Correlation presented the best performances (see Figure 1) for all the tested average synaptic connectivity degree (from 5 to 55 connections for each neuron).

Second we assessed the statistical significance of connections extracted through the aforementioned algorithms from electrophysiological data. Using “shuffling” techniques devised in [5], we implemented a reliable threshold-independent test, model free and not linked to any particular initial assumptions (i.e., choice of data distribution).

Finally, applying the validated methods, we obtained functional mapping of biological *in vitro* models of cortical networks and we extracted relevant topological features.
